# Effect of ridge preservation at molar extraction sites with severe periodontitis on the Schneiderian membrane thickness changes: a pilot study

**DOI:** 10.1186/s12903-021-01546-8

**Published:** 2021-04-12

**Authors:** Yiping Wei, Bo Zhang, Gang Yang, Tao Xu, Wenjie Hu, Kwok-Hung Chung

**Affiliations:** 1 Department of Periodontology, Peking University School and Hospital of Stomatology, National Clinical Research Center for Oral Disease, National Engineering Laboratory for Digital and Material Technology of Stomatology, Beijing Key Laboratory of Digital Stomatology, 22 Zhongguancun Avenue South, Haidian District, Beijing, 100081 People’s Republic of China; 2 Department of Stomatology, Beijing Friendship Hospital, Capital Medical University, Beijing, People’s Republic of China; 3 Department of Emergency, Peking University School and Hospital of Stomatology, National Clinical Research Center for Oral Disease, National Engineering Laboratory for Digital and Material Technology of Stomatology, Beijing Key Laboratory of Digital Stomatology, Beijing, People’s Republic of China; 4 Department of Restorative Dentistry, University of Washington, Seattle, WA USA

**Keywords:** Bone grafting, Cone-beam computed tomography, Mucosal thickening, Tooth extraction, Periodontitis

## Abstract

**Background:**

Information regarding the reaction of bone augmentation in terms to sinus mucosa thickness of periodontally compromised molar extraction sites is limited. This retrospective study aimed to analyze the effect of ridge preservation procedures following the extraction of molars with severe periodontitis on the healing pattern of adjacent maxillary sinus mucosal membranes.

**Methods:**

Thirty-one periodontally compromised maxillary molar teeth either receiving ridge preservation (test group, n = 20) or undergoing spontaneous healing (control group, n = 11) were investigated. Cone-beam computed tomography (CBCT) scanning was performed before the extraction procedure and repeated 6 months later. The mucosa thickness (MT) of the adjacent periodontally compromised molar tooth was measured from CBCT images before tooth extraction and after 6 months of healing at nine assigned measurement points. The data were analyzed at α = 0.05.

**Results:**

The prevalence of pre-extraction maxillary sinus mucosal thickening was 60.0% and 63.6% in the test and control groups, respectively. The average MT of the thickened sinus mucosa before tooth extraction was 3.78 ± 2.36 mm in the test group and 4.63 ± 3.20 mm in the control group (*P* = 0.063). The mean mucosal thickening reductions in the thickened MT subjects after 6 months of healing were 2.20 ± 2.05 mm (test group) and 2.64 ± 2.70 mm (control group), *P* = 0.289. The differences of MT between the time prior to extraction and after 6 months of healing were statistically significant within both groups (*P* < 0.05).

**Conclusions:**

Following extraction of molars with severe periodontitis, a reduction in swelling of the Schneiderian membrane has been observed regardless of the addition of a DBBM socket graft. However, a mucosal thickness > 2 mm was still frequently observed.

## Background

Periodontitis is a chronic inflammatory disease initiated by colonization of the microbial biofilm, which causes destruction of the ligament and alveolar bone supporting the teeth [1]. In comparison with other tooth types, maxillary molars have an increased susceptibility to periodontal diseases due to anatomic factors such as presence of furcations, concavities on the root surfaces, and multiple root prominence areas [2]. The spread of infections arising from posterior maxillary teeth to the maxillary sinus is facilitated by their close anatomical relationship. Periodontitis has been reported to be one of the principal causes of odontogenic maxillary sinusitis and the subsequent Schneiderian membrane thickening associated with it [3–6]. Ren et al. [5] demonstrated the relationships between mucosal thickening and periodontal pathologies using odds ratios. An odds ratio of 4.62 was obtained for patients with severe periodontal bone loss and increased mucous membrane thickening. Zhang et al. [3] reported alveolar bone loss and residual alveolar bone height were significantly associated with maxillary sinus mucosal thickening. Radiographically, sinus mucosal thickening of more than 2 mm is considered pathological [7–11].

Maxillary molars affected by severe periodontitis usually have a poor prognosis, ultimately leading to tooth loss. However, alveolar ridge resorption following tooth extraction may result in insufficient remaining bone support for dental implant placement [12, 13]. Ridge preservation is defined as any procedure that takes place immediately after tooth extraction to preserve ridge volume within the skeletal envelope that exists at the time of extraction [14]. The effectiveness of ridge preservation procedures in minimizing vertical dimensional change along with reducing the need for subsequent sinus augmentation in the posterior maxilla has been reported [15–17]. Moreover, the decrease in the vertical loss of ridge dimension is the result of a combination of reducing sinus pneumatization and crestal bone resorption [15, 16, 18].

Only a few articles have been published so far to evaluate the relationship between changes in the mucous membrane thickness of maxillary sinus from extractions [19, 20]. Yoo et al. [20] investigated the membrane thickness before implant placement in relation to the cause of extraction, but the data regarding membrane thickness prior to extraction were missing. Hsu et al. [19] assessed 14 odontogenic infected maxillary sinuses and found that thickened membrane diminished after about 3 months spontaneous healing following tooth extraction. However, there was no evidence available to identify the effect of ridge preservation at periodontally compromised molar extraction sites on adjacent maxillary sinus mucous membrane thickening. Pommer et al. [21] found that sinus membrane thickness increased significantly before versus 4 to 6 months after sinus floor augmentation via a lateral approach, using 50% autologous and 50% deproteinized bovine bone mineral. Quirynen et al. [22] indicated a transient swelling of the Schneiderian membrane despite a minimal invasive transcrestal sinus floor elevation. Until now, it is unclear whether the biomaterial played a role in swelling response of the membrane.

Therefore, the aim of this retrospective study was to evaluate the effect of ridge preservation at maxillary molar extraction sockets with severe periodontitis on the thickness of the Schneiderian membrane by cone beam computed tomography (CBCT). The null hypothesis was that no differences would be found between ridge preservation and spontaneous healing at periodontally compromised molar extraction sites in the reduction of thickness of the adjacent maxillary sinus mucosa.

## Methods

### Study population, inclusion and exclusion criteria

This retrospective study was conducted based on dental records and radiographs obtained from patients who were initially recruited for a prospective clinical trial (registered at Chinese Clinical Trial Registry, ChiCTR-ONN-16009433) involving ridge preservation from January 2015 to December 2018. Patients with a maxillary molar with severe periodontitis who planned to have subsequent implant-support crown rehabilitation were collected. From chart record analysis, patients who had undergone an extraction of a single maxillary molar with available pre- and post-extraction CBCT scans were identified. The project was conducted in accordance with the World Medical Association Declaration of Helsinki and approved by local ethics committee (Approval Number: PKUSSIRB-201946078). The subjects and CBCT images were selected only if they fulfilled the following criteria: (1) maxillary molar extraction due to severe periodontitis, teeth with perio-endo lesions only originating from periodontitis; (2) good quality images with the occlusal plane parallel to the floor; (3) at least one adjacent tooth at the proximal region; (4) no signs of air fluid levels and complete opacification of the sinus indicating acute sinusitis; (5) non-smoking status. Exclusion criteria were as follows: (1) history of maxillary sinusitis; (2) seasonal or pollen allergy reaction history; (3) patients with nasal congestion, a runny nose, fever, and any other nasal symptoms within the last 3 months at taking CBCT; (4) dental caries, cracked lesions, existed dental fillings, or root canal treatment in the posterior maxillary teeth; (5) membrane exposures/perforations at time of extraction; (6) maxillary mucosal cyst or bony septum; (7) history of periodontal surgery in the maxillary posterior region; (8) sinus surgery; (9) pregnancy and lactation.

### Surgical protocol

Clinical parameters, including probing depth, gingival recession and bleeding index, were measured by the same periodontist (WH) using a UNC-15 probe (Hu Friedy^®^, Chicago, IL, USA) before the tooth extraction procedure. Clinical attachment loss was calculated. Following administration of local anesthesia, the unsalvageable tooth was extracted atraumatically, and then full-thickness flaps were elevated buccally and lingually for exposure of only 2 mm of the alveolar bone crest of the socket. Sockets were meticulously debrided to remove all granulation tissue, then irrigated with sterile saline solution. Subjects were assigned into either a ridge preservation group or a spontaneous healing group according to their initial treatment plan. Extraction sockets in the ridge preservation subjects were filled with deproteinized bovine bone mineral (Bio-Oss^®^, Geistlich Pharma AG, Wolhusen, Switzerland) and covered by a bioabsorbable porcine collagen membrane (Bio-Gide^®^, Geistlich Pharma AG, Wolhusen, Switzerland). The extraction site was then covered with medical collagen sponge (Wuxi BIOT), which does not require primary soft tissue closure. A cross-mattress suture secured the collagen sponge in place. Sockets in the spontaneous healing group were filled with blood clots with no graft materials, and 4–0 silk sutures were used to stabilize blood clotting. No attempt was made for primary closure. All procedures were performed by the same periodontist (WH). Prescriptions post operatively for antibiotic (Amoxicillin 500 mg, t.i.d.) for 7 days and analgesics (ibuprofen 300 mg, b.i.d.) for 3–5 days were provided. Avoiding brushing the surgical area and mouth rinsing with 0.12% chlorhexidine twice daily for 2 weeks was instructed.

### Cone beam computed tomography (CBCT) measurements

CBCT scans were taken before the extraction procedure and repeated 6 months later using the same imaging unit (NewTom VG, Aperio Services, Italy) at a slice thickness of 0.125 mm, field of view size 8 × 8 cm, and pixel size of 0.125 mm; exposure parameters were 15 s, 110 mkVp, and 12–17 mAs.

Alveolar bone loss and minimum residual alveolar bone height (minRABH) measured in the CBCT images taken before extraction using the same methods as described by Zhang et al. [3]. The alveolar bone loss of each tooth was calculated as a maximal percentage of normal alveolar bone height. Normal alveolar bone height was determined as the distance from 2 mm below the cemento-enamel junction to the tip of the root [23]. Minimum residual alveolar bone height (minRABH) defined as the shortest vertical distance from the most apical alveolar bone of the molar to the bony edge of the maxillary sinus.

**Fig. 1:**
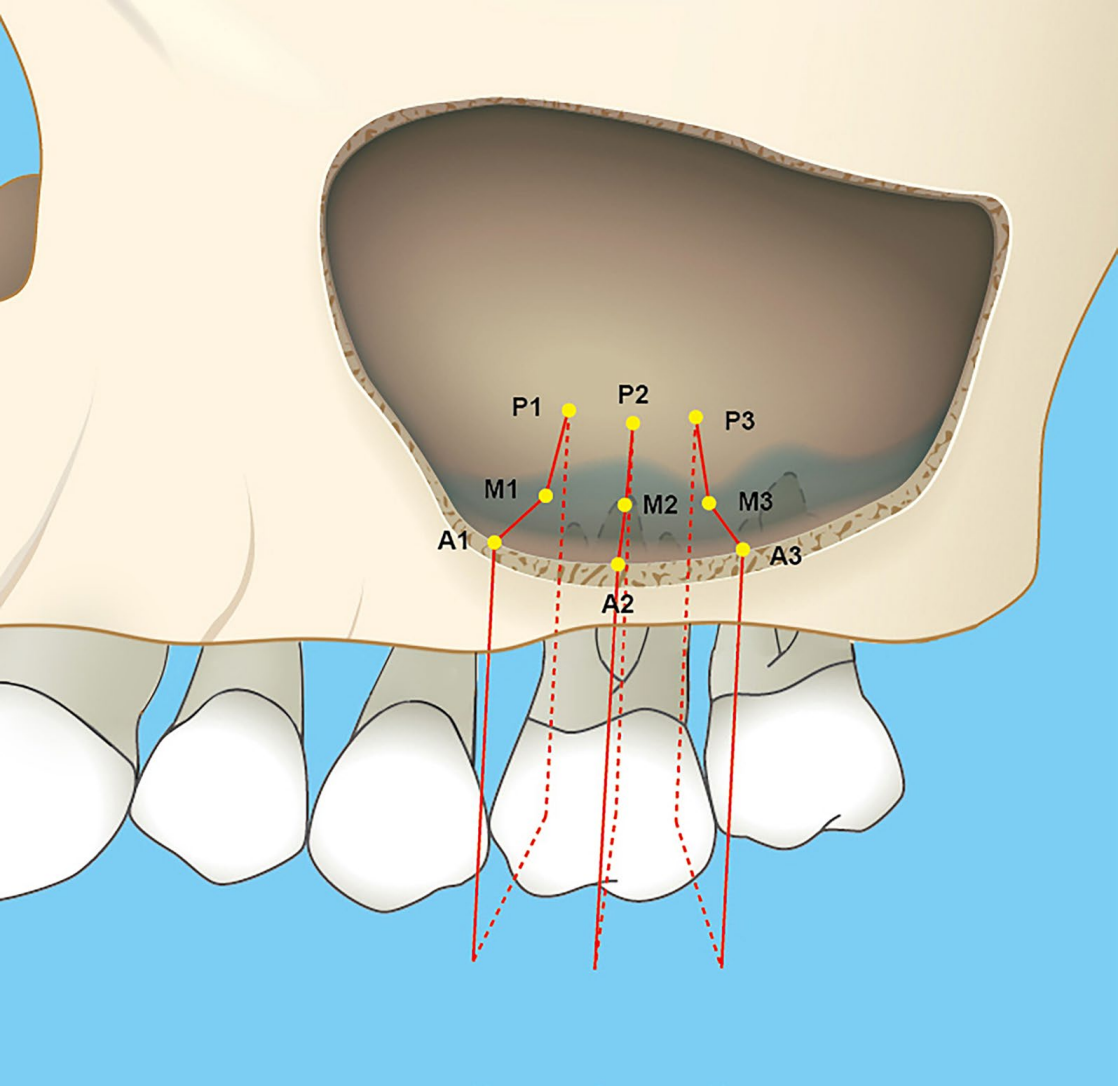
Schematic overview of the nine measurement points in the evaluated maxillary sinuses of a representative upper first molar before tooth extraction. A1, M1 and P1 for the measurement of MT at coronal slices in mesial region (the anterior wall, the sinus floor, the posterior wall). A2, M2 and P2 at coronal slices in central region. A3, M3 and P3 at coronal slices in distal region

**Fig. 2:**
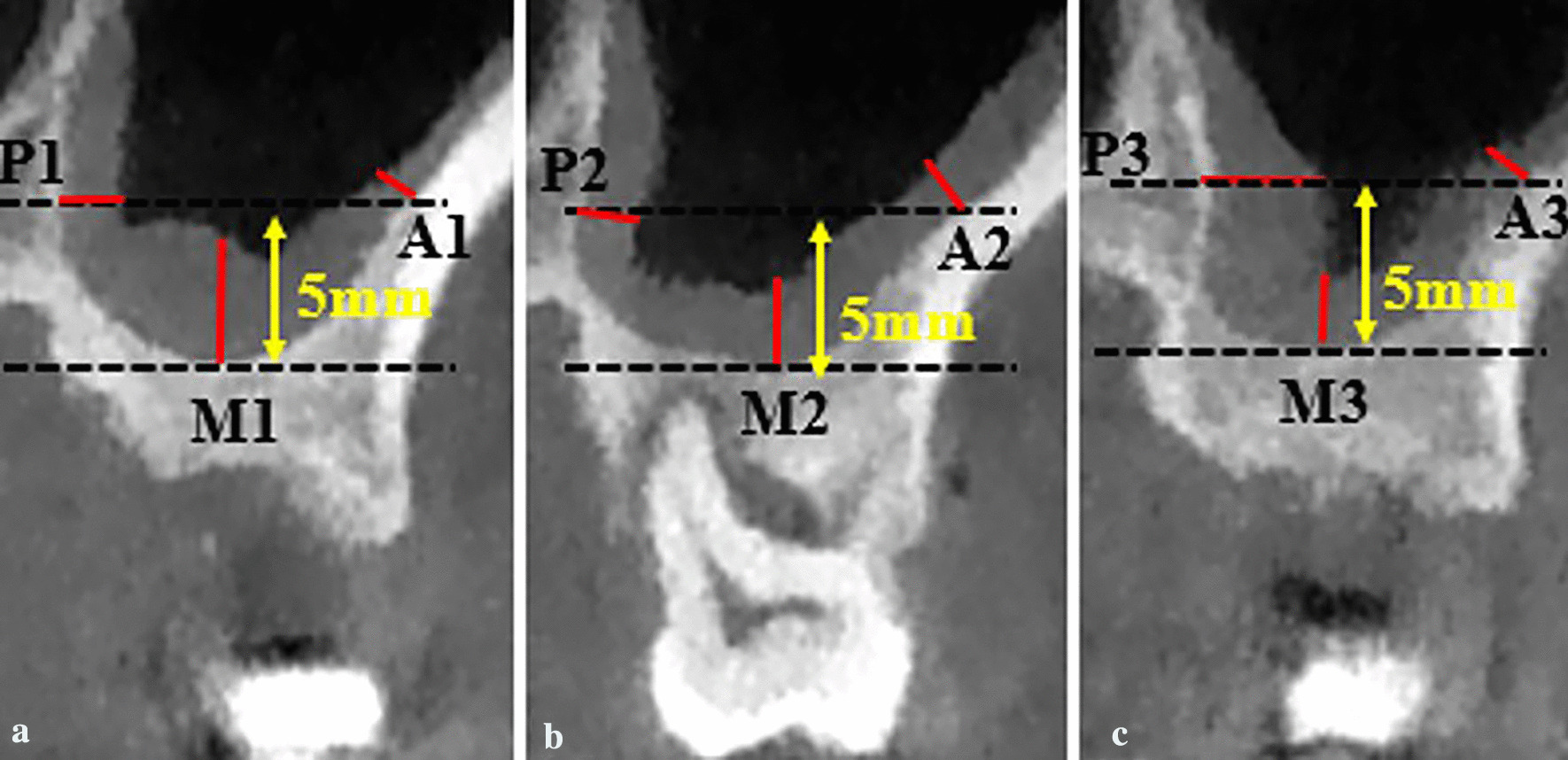
Landmarks for the measurement of MT in coronal CBCT slices. The measurement points of anterior wall and posterior wall of maxillary sinus were all obtained at 5 mm from sinus floor. **a** the mesial region of pre-extraction CBCT image; **b** the central region of pre-extraction CBCT image; **c** the distal region of pre-extraction CBCT image

Two sets of DICOM (Digital Imaging and Communications in Medicine) data were generated and transferred to volumetric imaging software (Mimics 17.0, Materialise). The two data sets were aligned by the palatal vault of the maxilla in three planes (coronal, axial, and sagittal). Two identically oriented scans along the long axes of the teeth in both sagittal and coronal planes were produced. The thickness of the mucous membrane was measured at the mesial, central, and distal coronal sections of extraction sockets (Fig. 1). In each coronal section, mucosal thickness (MT) perpendicular to the underlying bone was measured at three points: (1) the lowest point of the sinus floor (M), (2) the point of anterior wall of maxillary sinus obtained at 5 mm from sinus floor (A), (3) the point of posterior wall of maxillary sinus obtained at 5 mm from sinus floor (P) (Fig. 2); the mean of the nine values was used as a reference. Mucosal thickening was considered to be present when the thickness of the sinus mucosa was > 2 mm.

A research investigator (BZ) made all the radiographic measurements to the nearest 0.01 mm. Intra-examiner calibration was conducted by measuring the variables twice with an interval of 14 days on 10 randomly selected CBCT images prior to the final data measurement. The intraclass correlation coefficient was determined to be 0.995.

### Statistical analysis

Data were analyzed by using SPSS 20.0 software (SPSS Inc., Chicago, IL, USA). Descriptive data for all parameters were performed, and the normality of the data was tested by the Shapiro–Wilk test. A paired samples *t *test, or a Wilcoxon signed rank test for any data that were not normally distributed, was used to evaluate within group differences between pre-extraction and 6 months post-extraction. Student’s *t *test or Mann–Whitney U test was performed to compare parameters between test and control groups. The Chi-square test was used for the categorical variables. All statistical tests were two-tailed with a significance level of 0.05.

## Results

A total of 31 patients (contributing 31 teeth) were included for the analysis. Of these, 20 patients (contributing 20 teeth) received ridge preservation procedures to serve as the test group. The median age was 54 years (range: 25 to 61 years), and there were 11 males and 9 females. The other 11 patients (contributing 11 teeth) with spontaneous healing sites served as controls. The median age was 51 years (range: 32 to 62 years), and there were 8 males and 3 females. CBCT images with mucosal thickening were observed in 12 sinuses of the test group and 7 sinuses of the control group before tooth extraction. The prevalence of maxillary sinus mucosal thickening was 60.0% and 63.6% in the test and control groups, respectively. Demographic and clinical characteristics are presented in Table 1. No statistically significant differences were observed between the test and control groups (*P* > 0.05).

**Table 1:** Demographics and clinical characteristics of test and control groups

	Test Group		Control Group		*P* values
	Normal MT	Thickened MT	Normal MT	Thickened MT	
Age (years)					
Median (range)	52.0 (43–61)	54.5 (25–59)	52.5 (45–62)	47.0 (32–60)	0.583^a^
Gender					
Male	3	8	2	6	
Female	5	4	2	1	0.452^b^
Number of teeth	8	12	4	7	
Tooth position					
Maxillary first molar	5	7	4	6	
Maxillary second molar	3	5	0	1	0.106^b^
Probing depth (mm)					
Mean ± SD	5.5 ± 1.6	6.2 ± 1.3	6.6 ± 1.0	5.3 ± 1.6	0.595^c^
Clinical attachment loss (mm)					
Mean ± SD	7.6 ± 1.5	8.2 ± 1.7	8.7 ± 2.1	7.9 ± 1.9	0.512^c^
Gingival recession (mm)					
Mean ± SD	2.3 ± 1.1	2.3 ± 2.1	3.1 ± 2.5	2.8 ± 2.2	0.444^c^
Bleeding index					
Mean ± SD	3.2 ± 1.0	3.7 ± 0.4	3.8 ± 0.3	3.2 ± 0.7	0.498^c^
Alveolar bone loss (%)					
Median (range)	70.4 (62.0–100)	83.7 (70.4–100)	77.2 (65.4–100)	85.1 (68.9–100)	0.683^a^
minRABH (mm)					
Median (range)	1.26 (0.22–5.25)	0.59 (0.10–2.28)	1.06 (0.53–5.73)	0.65 (0.13–2.67)	0.729^a^
Healing time (months)					
Median (range)	6.0 (5–10)	6.0 (5–8)	7.0 (5–10)	6.0 (4–11)	0.555^a^

*P* value in the last column are intergroup *P* values comparing test and control groups

MT, mucosal thickness; minRABH, minimum residual alveolar bone height

^a^Mann–Whitney U test

^b^Chi‐square test

^c^Student’s *t *test

Table 2 gives values for MT in normal MT test groups (n = 8) and normal MT control groups (n = 4), as well as mean changes from pre-extraction to 6 months post-extraction. No statistically significant differences were observed (*P* > 0.05).

**Table 2:** MT in normal MT test groups (n = 8) and normal MT control groups (n = 4) and mean changes from pre-extraction to 6 months post-extraction (mm; mean ± SD)

	Normal MT in test group	Normal MT in control group	*P* value
Pre-extraction	1.35 ± 0.83	1.54 ± 0.93	0.330
Post-extraction	1.15 ± 0.83	1.32 ± 0.86	0.256
Changes	0.20 ± 0.47	0.22 ± 0.66	0.355
*P* value	0.106	0.338	

*P *value in the last row are intragroup *P* values comparing changes of mucosal thickness from pre-extraction to 6 months post-extraction by paired samples *t *test

*P *value in the last column are intergroup *P* values by Student’s *t *test

MT, mucosal thickness

**Fig. 3:**
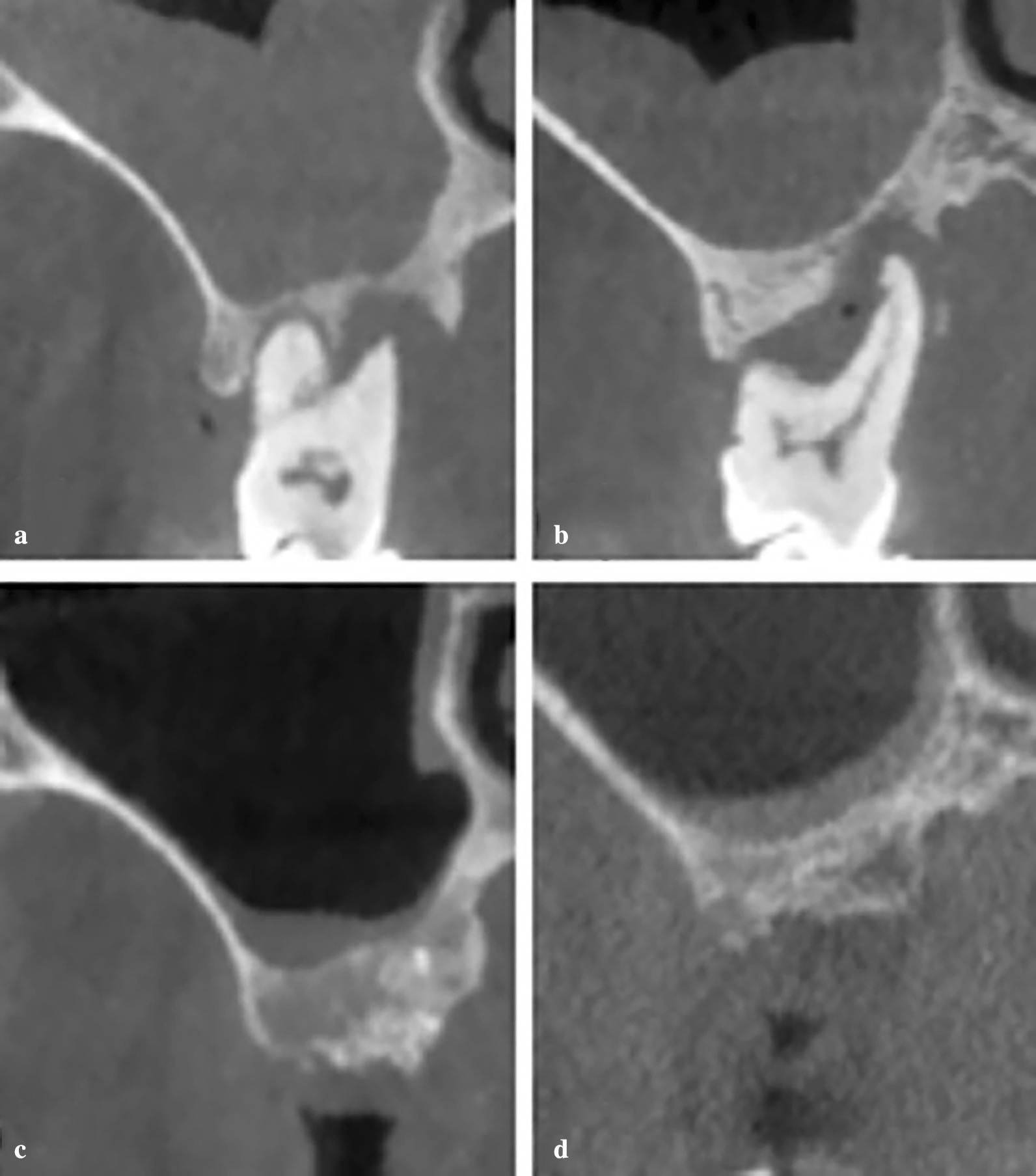
Representative images of coronal-sectional CBCT scans from sinuses with thickened MT in the pre- and 6 months post-extraction of the test and control groups. **a** Pre-extraction maxillary sinus with thickened MT of the test group. **b** Pre-extraction maxillary sinus with thickened MT of the control group; **c** 6 months post-extraction of the test group; **d** 6 months post-extraction of the control group

Regarding the MT of maxillary sinus membrane before tooth extraction, the mean MT values was 3.78 ± 2.36 mm in the thickened MT test group (n = 12) and 4.63 ± 3.20 mm in the thickened MT control group (n = 7) (*P* = 0.063) (Table 3). After a 6-month healing period, the MT reduced to 1.58 ± 0.93 mm and 1.99 ± 1.54 mm in the thickened MT test group and control group, respectively, which were not significantly different from normal MT group. The mean mucosal thickening reduction as assessed by CBCT measurement was 2.20 ± 2.05 mm and 2.64 ± 2.70 mm, respectively. The difference between pre-extraction MT and post-extraction MT was statistically significant (*P* < 0.05) (Fig. 3).

**Table 3:** MT in thickened MT test groups (n = 12) and thickened MT control groups (n = 7) and mean changes from pre-extraction to 6 months post-extraction (mm; mean ± SD)

	Thickened MT in test group	Thickened MT in control group	*P* value
Pre-extraction	3.78 ± 2.36	4.63 ± 3.20	0.063
Post-extraction	1.58 ± 0.93	1.99 ± 1.54	0.329
Changes	2.20 ± 2.05	2.64 ± 2.70	0.289
*P* value	< 0.001*	< 0.001*	

MT, mucosal thickness

*P *value in the last row are intragroup *P* values comparing changes of mucosal thickness from pre-extraction to 6 months post-extraction by paired samples *t *test

*P *value in the last column are intergroup *P* values by Student’s *t *test

*Statistically significant difference (*P* < 0.05)

The detailed MT at nine measurement points at both pre-extraction and 6-month post-extraction are presented in Table 4. There was a wide range of MT before extraction, with a maximum value of 12.74 mm and a minimum value of 0.45 mm in the thickened MT test group, and a range of 0.41–12.59 mm in the thickened MT control group. Overall, no statistically significant differences in mean MT were observed among the nine measurement points in the two groups. Before tooth extraction, the mean MT values was 3.15 ± 1.75 mm, 4.83 ± 2.57 mm and 3.38 ± 2.39 mm for anterior wall regions (A1, A2 and A3), sinus floor regions (M1, M2 and M3) and posterior wall regions (P1, P2 and P3) in the thickened MT test group. In the thickened MT control group, the mean MT values was 3.83 ± 2.26 mm, 6.12 ± 3.31 mm and 3.86 ± 3.46 mm for anterior wall, sinus floor and posterior wall regions, respectively. Higher MT values were observed for sinus floor regions compared with those for anterior wall and posterior wall regions in two groups (*P* < 0.05). After 6 months post-extraction, MT has decreased for all three regions with the values of 1.36 ± 0.82 mm, 1.93 ± 0.96 mm and 1.45 ± 0.92 mm for anterior wall, sinus floor and posterior wall regions in the thickened MT test group, and with the values of 1.54 ± 0.83 mm, 2.82 ± 1.93 mm and 1.60 ± 1.39 mm in the thickened MT control group respectively. Similarly, higher MT values were also observed for sinus floor regions compared with those for anterior wall and posterior wall regions in two groups (*P* < 0.05).

**Table 4:** MT obtained at nine measurements in thickened MT test groups (n = 12) and thickened MT control groups (n = 7) and mean changes from pre-extraction to 6 months post-extraction (mm)

Groups	Mesial			Central			Distal		
	A1	M1	P1	A2	M2	P2	A3	M3	P3
*Pre-extraction*									
Thickened test									
Median	2.89	4.17	2.56	3.02	4.11	2.99	3.17	4.59	2.57
Range	1.31–9.87	2.26–12.74	1.53–9.77	0.45–5.50	1.81–10.26	0.80–10.47	0.91–4.68	2.01–9.65	0.86–5.52
Thickened control									
Median	2.90	4.91	4.16	4.08	6.92	3.03	4.38	7.55	2.65
Range	2.10–6.87	4.31–8.45	0.41–9.28	2.07–8.48	3.86–12.59	0.41–9.05	1.73–8.72	0.18–12.5	0.25–10.3
* P* value	0.827	0.079	1.000	0.803	0.134	1.000	0.248	0.566	0.526
*Post-extraction*									
Thickened test									
Median	1.27	1.72	1.68	1.06	2.37	1.11	1.13	1.71	0.85
Range	0.79–2.72	0.76–4.18	0.91–4.50	0.23–3.53	0.65–3.16	0.26–3.21	0.25–2.90	0.76–3.88	0.59–1.48
Thickened control									
Median	0.86	2.21	1.13	1.27	3.00	1.64	1.51	2.49	1.51
Range	0.64–3.24	1.08–6.37	0.26–2.77	0.81–3.01	0.80–5.41	0.18–5.74	0.70–1.96	0.20–5.52	0.41–2.57
* P* value	0.441	0.510	0.115	0.717	0.322	0.354	0.732	0.536	0.582
*Changes (mean ± SD)*									
Thickened test									
Median	1.47	2.25	0.68	1.64	3.51	3.95	1.47	3.98	1.85
Range	0.41–8.6	1.05–8.56	0.27–8.02	0.13–5.27	2.54–7.22	0.69–8.45	0.10–3.06	1.19–6.07	0.94–4.04
Thickened control									
Median	2.00	3.23	1.39	1.07	3.06	1.49	2.42	2.72	1.14
Range	0.55–5.12	0.18–6.24	0.15–8.15	0.75–7.67	1.64–9.59	0.10–7.35	0.83–6.90	0.02–8.17	0.16–8.36
* P* value	0.510	1.000	0.320	0.674	0.533	0.743	0.439	0.935	0.661

Mann–Whitney U test was performed to compare data between groups

MT, mucosal thickness

Implants were placed in a total of 31 patients at 6 months postoperatively. No post-operative complications were recorded at any included site. Additional sinus augmentation was performed in 25% (15 out of 20 subjects) of the patients in the test group and 63.7% (7 out of 11 subjects) of those in the control group. The crestal approach for sinus floor elevation was performed in three cases, and the lateral window approach was performed in two cases in the test group, while three and four in the control group, respectively.

## Discussion

This retrospective study aimed to evaluate the effect of ridge preservation following extraction of molars with severe periodontitis on Schneiderian membrane thickness of the maxillary sinus. Results revealed that a similar reduction in the mucosal thickening of the maxillary sinus would be observed after ridge preservation compared to spontaneous healing. Therefore, the null hypothesis was accepted. In the present study, the prevalence of maxillary sinus mucosal thickening was 60.0% and 63.6% in the test and control groups respectively, which is consistent with previously published studies [3–6]. Zhang et al. [3] reported that the prevalence of mucosal thickening was 75.5% for patients with severe alveolar bone loss. Periodontal infection may reach the sinus by the spread of microorganisms and their pathogenic products through vascular and lymphatic systems or via a direct diffusion through porous maxillary bone, affecting the sinus mucosa [24].

For a more in-depth understanding, MT of the maxillary sinus was assessed at nine different points. Higher MT values were observed for sinus floor regions compared with those for anterior wall and posterior wall regions. The average MT of the thickened sinus mucosa was 3.78 ± 2.36 mm in the test group and 4.63 ± 3.20 mm in the control group, similar to a previous study that found mean MT prior to extraction was 4.53 ± 2.46 mm [19]. After extraction, MT decreased by 2.20 mm and 2.64 mm in the thickened MT test group and control group, respectively, with a median healing time of 6 months, and these differences between pre-extraction and post-extraction were statistically significant. Hsu et al. [19] demonstrated that the mean MT reduction was 3.28 mm in 14 maxillary sinuses with membrane thickening, and thickened Schneiderian membrane resolution was observed by 2.80 ± 1.37 months post-extraction. These dissimilarities may be due to differences in the reasons for extraction; most thickened mucosa in Hsu’s study were in the acute stage of infection. Yoo et al. [20] discovered that the thickened Schneiderian membrane needed more than 12 months to recover to a thickness of < 2 mm for patients requiring extraction as a result of periodontal disease. In accordance with this study, an MT value of more than 2 mm was still found at some measurement points after 6 months post-extraction.

Although studies of infected extraction sockets are sparse, it is known that healing dynamics in compromised extraction sockets are different than those observed for extraction sockets unaffected by periodontitis [25–27]. Reconstruction of alveolar ridge volume in maxillary molar extraction sockets affected by severe periodontitis presents clinical challenges. Several studies indicated that ridge preservation in the posterior maxilla maintained the vertical bone height more efficiently and resulted in less need for sinus augmentation procedures compared to spontaneous healing [15–17]. Cha et al. [15] reported that implant placement without any additional sinus augmentation procedure was needed in 42.9% of ridge preservation cases, whereas in all of the subjects (100% of the cases) in the spontaneous healing group, an additional augmentation procedure was performed. In this current study, implant placement with additional sinus augmentation procedure was performed in 25.0% of test group cases, while 63.7% of the cases in the control group. Approximately 10.0% (2/20) of the test group patients received a lateral sinus augmentation, whereas the control group showed 36.4% (4/11). Alveolar ridge preservation was associated with less invasive (lateral window versus transcrestal approach) sinus augmentation techniques, which is in accordance with previous findings [28]. The major disadvantages of sinus floor augmentation included the longer treatment time, more surgical sittings, greater financial costs, and patient-reported outcome measures, as well as other factors [29]. Perforation of the maxillary sinus mucosa is one of the most common complications of the sinus augmentation procedure [30]. MT is an important factor related to sinus membrane perforation [31]. Therefore, the precise assessment of MT is crucial prior to surgery. Nevertheless, the effect of ridge preservation on maxillary sinus mucous membrane thickening is unknown. Removing the source of infection is the most important treatment for odontogenic sinus membrane thickening. Likewise, meticulous debridement and removal of the infectious source were the keys to successful ridge preservation in infected sockets. In this current study, decreases in MT were not significantly different between thickened MT test groups and control groups, which revealed that conventional debridement adequately removed the source of infection in ridge preservation at infected molar sites.

The effect of alveolar ridge preservation in terms of dimensional changes in the alveolar crest was not reported in the present study, since the objective of this research was only to assess the changes of the thickness of the Schneiderian membrane after ridge preservation.

One limitation of the present study was the small sample size. Moreover, this was a retrospective study. The thickened MT might have subsided before the first CBCT examination post-extraction; however, we have no available data. Another limitation was the lack of consideration of histological evaluation, which is the gold standard for the evaluation of the quality of MT. Although CBCT examiations have been widely used in many studies, the limitation of CBCT studies should not be ignored. Monje et al. [32] reported that CBCT assessment was higher than the histological examination in MT. Moreover, Brullmann & Schulze [33] indicated that higher accuracy than 0.5 mm cannot be expected in CBCT clinical application.

## Conclusions

Within the limitations of this study, it can be concluded that following extraction of molars with severe periodontitis, a reduction in swelling of the Schneiderian membrane has been observed irrespective of the addition of a DBBM socket graft. However, a mucosal thickness > 2 mm was still frequently observed.

## Data Availability

The datasets used during the current study are available from the corresponding author on reasonable request.

## Abbreviations

CBCT: Cone-beam computed tomography
MT: Mucosa thickness
minRABH: Minimum residual alveolar bone height
